# A Thrombosed Popliteal Artery Aneurysm Masquerading as a Benign Soft-Tissue Mass: A Case Report Emphasizing the Role of Multimodal Imaging

**DOI:** 10.3390/diagnostics16162513

**Published:** 2026-08-10

**Authors:** Po-Yin Shen, Yi-Hsueh Liu, Po-Chao Hsu, Wei-Ting Wu, Ke-Vin Chang, Levent Özçakar

**Affiliations:** 1 Department of Orthopaedics, Kaohsiung Municipal Siaogang Hospital, Kaohsiung 812, Taiwan; vanness068@gmail.com; 2 Department of Orthopaedics, Kaohsiung Medical University Hospital, Kaohsiung Medical University, Kaohsiung 807, Taiwan; 3 School of Medicine, College of Medicine, Kaohsiung Medical University, Kaohsiung 807, Taiwan; liuyh@kmu.edu.tw (Y.-H.L.); pochao.hsu@gmail.com (P.-C.H.); 4 Division of Cardiology, Department of Internal Medicine, Kaohsiung Medical University Hospital, Kaohsiung 807, Taiwan; 5 Department of Internal Medicine, Kaohsiung Municipal Siaogang Hospital, Kaohsiung 812, Taiwan; 6 Department of Physical Medicine and Rehabilitation, Community and Geriatric Research Center, National Taiwan University Hospital, Bei-Hu Branch, Taipei 108206, Taiwan; b40560@bh.ntuh.gov.tw; 7 Department of Physical Medicine and Rehabilitation, College of Medicine, National Taiwan University, Taipei 100233, Taiwan; 8 Center for Regional Anesthesia and Pain Medicine, Wang-Fang Hospital, Taipei Medical University, Taipei 110301, Taiwan; 9 Department of Physical and Rehabilitation Medicine, Hacettepe University Medical School, Ankara 06100, Turkey; lozcakar@yahoo.com

**Keywords:** popliteal artery, aneurysm, ultrasonography

## Abstract

Popliteal artery aneurysms (PAA) lose their pulsatility once thrombosed and may therefore be mistaken for a Baker cyst or a soft-tissue tumor. A 58-year-old male presented with a 6-month history of progressive right popliteal swelling and pain, accompanied by right foot paresthesia and diminished distal pulses for two months. The presence of accompanying neurovascular deficits shifted the diagnostic consideration from a benign soft-tissue lesion to a vascular disorder. In this patient, non-contrast imaging protocol integrating grayscale ultrasound, power Doppler, and magnetic resonance imaging (MRI) successfully identified a chronically thrombosed popliteal artery aneurysm, supporting a vascular rather than neoplastic etiology. While ultrasound cannot replace contrast-enhanced MRI for definitive tissue characterization, its real-time capability established a vascular etiology and guided subsequent diagnostic digital subtraction angiography with urgent endovascular stent-graft reconstruction. Furthermore, serial ultrasonographic surveillance at three and 18 months confirmed long-term stent-graft patency and favorable aneurysmal sac remodeling. Neurovascular deficits accompanying a popliteal mass should redirect the workup toward vascular imaging. This case underscores a critical diagnostic pitfall, demonstrating that a stepwise strategy combining real-time ultrasound and non-contrast MRI ensures rapid definitive management for complex popliteal masses while preserving the unique role of sonography in non-invasive follow-up.

**Figure 1:**
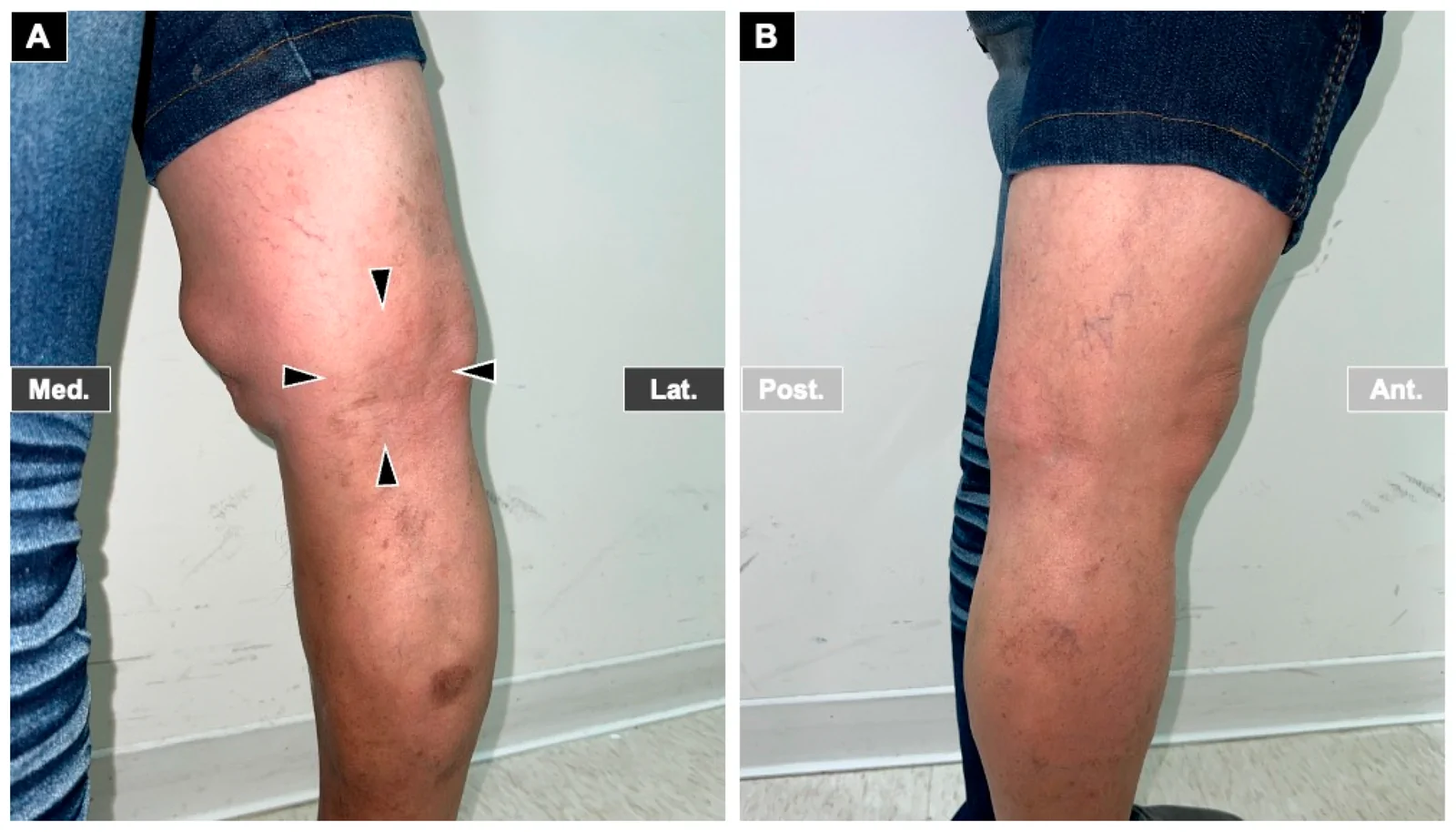
Clinical presentation of a right popliteal mass mimicking a benign soft-tissue lesion. Panels (**A**) (posterior oblique view) and (**B**) (lateral view) illustrate a 58-year-old male presenting with a 6-month history of progressive right popliteal pain and swelling, followed by right foot paresthesia for two months. His medical history included hypertension, hyperlipidemia, and a smoking history of more than 30 pack-years. He had no previous history of peripheral arterial disease (PAD). Physical examination reveals prominent focal swelling within the right popliteal fossa, characterized by a firm, mass-like fullness (panel (**A**), black arrowheads); notably, distal arterial pulses are severely diminished upon palpation. While isolated popliteal fullness in outpatient settings frequently prompts an initial diagnosis of Baker cyst or common benign soft-tissue mass [1,2,3], the atypical combination of neurological symptoms and compromised distal perfusion strongly indicates an underlying vascular pathology, such as popliteal artery aneurysm (PAA) or subsequent thromboembolism [2]. Abbreviations: Ant., anterior; Lat., lateral; Med., medial; Post., posterior.

**Figure 2:**
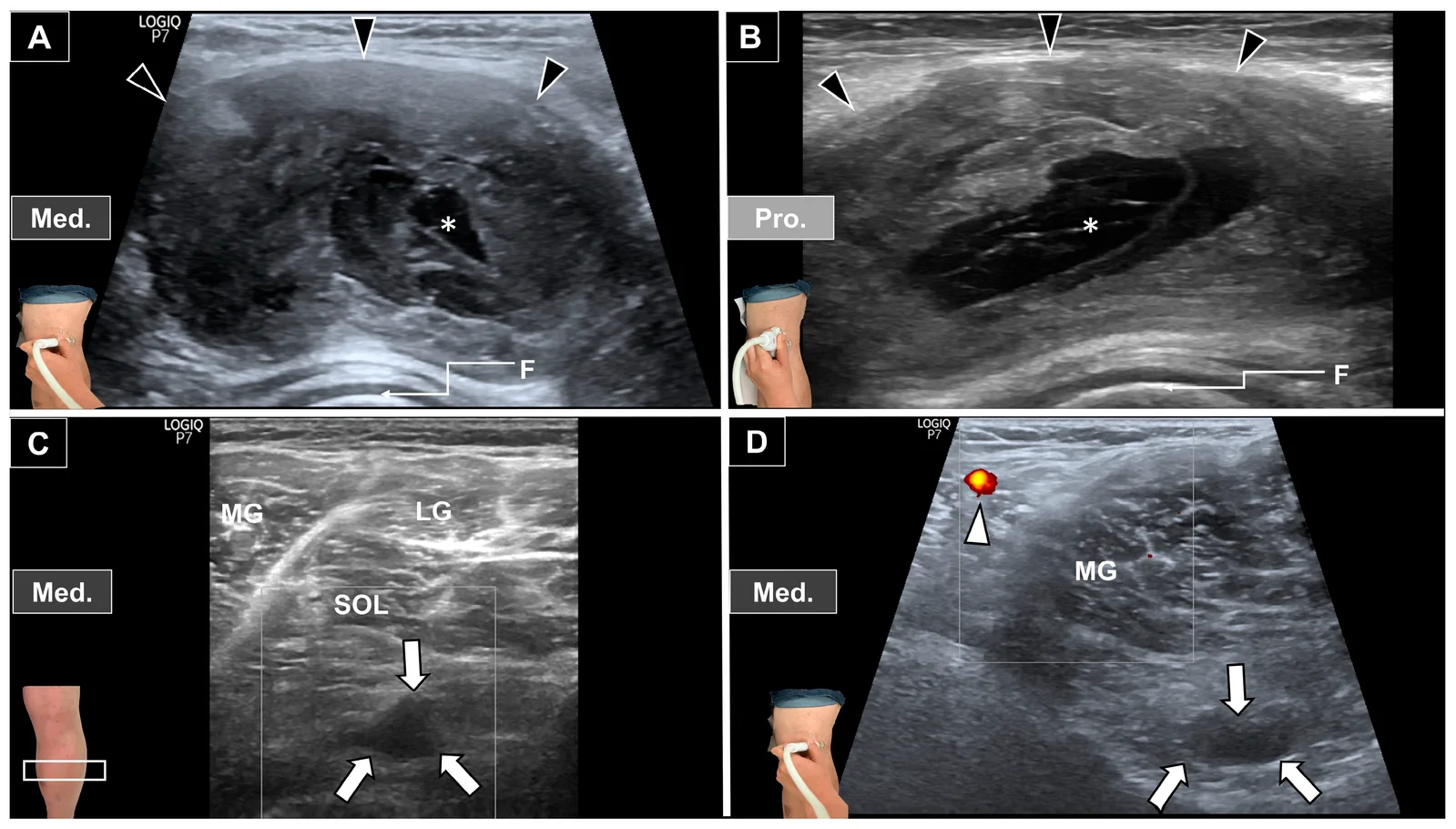
Grayscale and power Doppler ultrasonographic appearance of a chronically thrombosed popliteal artery aneurysm mimicking a soft-tissue mass. Panels (**A**,**B**) illustrate the short-axis and long-axis grayscale views, respectively. Both images reveal an expansile, markedly enlarged popliteal aneurysmal sac (black arrowheads) adjacent to the posterior femoral cortex (F). On the short-axis image, the thrombosed aneurysmal sac measured 4.57 cm × 3.14 cm. The sac was almost completely filled with a heterogeneous, laminated thrombus [4]. A localized hypoechoic region (*) within the thrombus matrix suggests asynchronous clot formation, with the variable echotexture potentially reflecting different stages of organization and lysis rather than a residual patent lumen. Panels (**C**,**D**) display power Doppler images obtained in the distal short-axis and long-axis planes. Panel (**C**) demonstrates occlusion of the distal popliteal artery (white arrows) within the intermuscular interval between the medial (MG) and lateral (LG) heads of the gastrocnemius muscle [1,2]. Panel (**D**) confirms the absence of detectable flow within the occluded distal segment (white arrows), while an adjacent collateral vessel with preserved flow is identified nearby (white arrowhead). This collateral supply likely explains why the limb remained viable, presenting with paresthesia and diminished distal pulses rather than acute limb ischemia. Ultrasound also narrowed the differential diagnosis at this stage. A Baker cyst was considered unlikely because the lesion was neither anechoic nor thin-walled and did not arise posteromedially from the gastrocnemius–semimembranosus bursa [1,3]. A solid soft-tissue neoplasm was likewise unlikely: sarcomas of comparable size typically demonstrate internal vascularity on power Doppler, whereas no internal flow was detectable here [5]. The absence of internal flow, the concentric laminated architecture, and direct continuity with the expected course of the popliteal artery together favor a chronically thrombosed aneurysm over a cystic or solid soft-tissue mass [1,2]. Ultrasound alone cannot exclude a hypovascular tumor, and MRI was therefore obtained to characterize the internal composition and narrow the differential further. Popliteal artery aneurysms are frequently bilateral and may coexist with abdominal aortic aneurysm [4]. The contralateral popliteal artery and the abdominal aorta were therefore also screened, and neither showed aneurysmal dilatation. Abbreviations: F, femur; LG, lateral head of gastrocnemius muscle; Med., medial; MG, medial head of gastrocnemius muscle; Pro., proximal.

**Figure 3:**
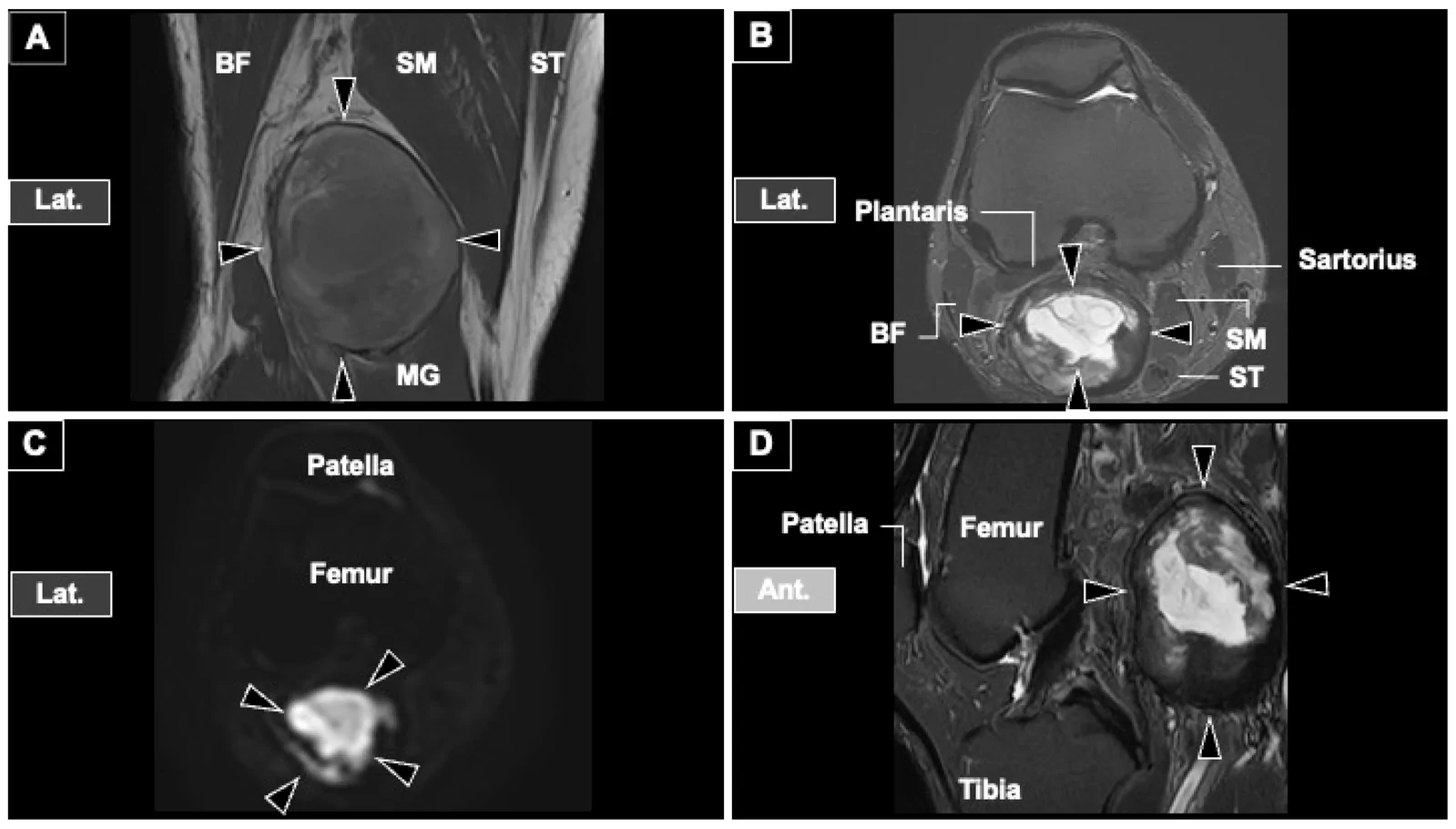
Non-contrast magnetic resonance imaging (MRI) features of a chronically thrombosed popliteal artery aneurysm and its differential diagnosis. Panel (**A**) (coronal T1-weighted image) shows a large, well-defined oval mass (black arrowheads) in the popliteal fossa. This lesion is situated beneath the biceps femoris (BF), semimembranosus (SM), and semitendinosus (ST) and cranial to the medial gastrocnemius (MG). Its intermediate-to-low internal signal with subtle concentric laminations reveals an organized intraluminal thrombus rather than a simple synovial cyst [1]. A Baker cyst, by contrast, shows homogeneous fluid signal intensity [1,5]. Panel (**B**) (axial T2-weighted short tau inversion recovery [STIR] view) reveals a well-circumscribed, heterogeneous mass (black arrowheads) within the central neurovascular compartment, displacing the BF laterally and the SM, ST, and plantaris medially. Although the T2/STIR hyperintensity could mimic a necrotic soft-tissue tumor or hematoma, the deep central location along the expected course of the popliteal artery favors a vascular origin [1,6]. Panel (**C**) (axial diffusion-weighted view [DWI]) demonstrates heterogeneous hyperintensity within the lesion core (black arrowheads). This finding can make diagnosis challenging because organized thrombi, subacute hematomas, and highly cellular soft-tissue tumors may exhibit overlapping signal characteristics on non-contrast MRI sequences. Completely thrombosed peripheral aneurysms have occasionally been misdiagnosed as soft-tissue sarcomas [6,7]. Panel (**D**) (sagittal T2-weighted STIR view) illustrates the longitudinal extent of the lesion (black arrowheads) following the anatomical trajectory of the popliteal artery. Contrast-enhanced sequences were not pursued. Grayscale ultrasound, power Doppler, ankle-brachial index (ABI) assessment, and non-contrast MRI collectively favored a vascular rather than neoplastic etiology. Although CT angiography is generally considered the preferred imaging modality for preoperative planning of popliteal artery aneurysms, non-contrast MRI was selected in this case because the primary diagnostic challenge was characterization of an atypical popliteal fossa mass and exclusion of a soft-tissue neoplasm. Furthermore, progressive ischemic symptoms and severely diminished distal pulses necessitated prompt digital subtraction angiography (DSA), which simultaneously confirmed chronic femoropopliteal occlusion and enabled immediate endovascular treatment with covered stent-graft deployment [4,8]. Abbreviations: Ant., anterior; BF, biceps femoris; Lat., lateral; MG, medial head of gastrocnemius muscle; SM, semimembranosus; ST, semitendinosus.

**Figure 4:**
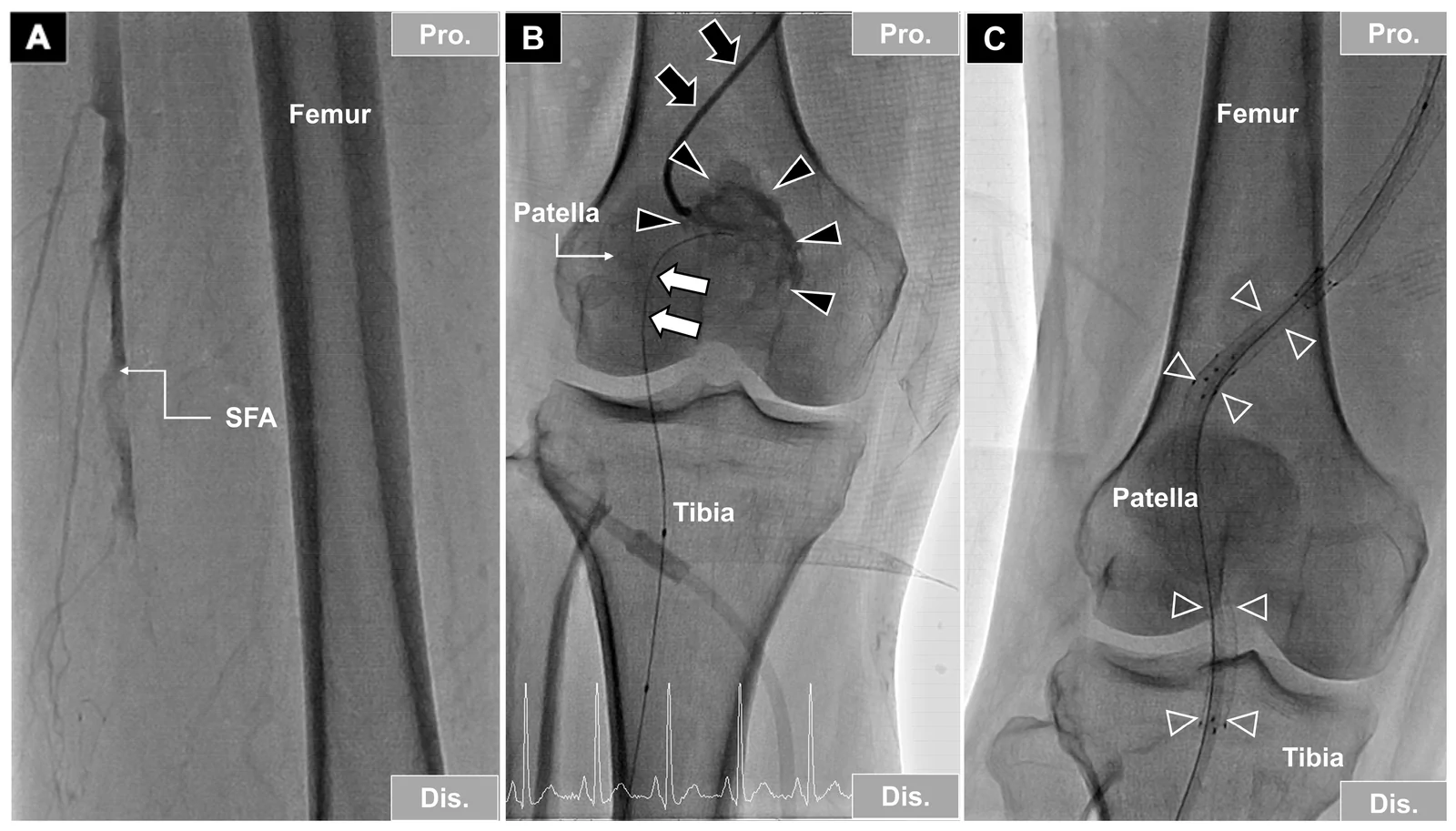
Digital subtraction angiography (DSA) and endovascular reconstruction of the thrombosed popliteal artery aneurysm. Panel (**A**) shows chronic total occlusion of the distal superficial femoral artery (SFA) with abrupt termination proximal to the thrombosed aneurysmal segment. The aneurysmal sac shows no contrast opacification (black arrowheads), which confirms its complete exclusion from active luminal flow. This lack of contrast opacification correlates with the heterogeneous, thrombus-filled sac observed on prior ultrasound and magnetic resonance imaging (MRI). Panel (**B**) shows an endovascular catheter (white-outlined black arrow) advanced antegrade from the SFA toward the occluded native popliteal artery (white arrows). Unlike non-invasive modalities, catheter-based angiography directly delineates the inflow obstruction, collateral networks, and distal runoff to guide endovascular reconstruction [4,8]. A further consideration was that open exposure would have required extensive dissection around the large, thrombosed sac. Endovascular repair was nevertheless selected in this patient, because the presentation was one of symptomatic chronic popliteal occlusion and the anatomy was suitable for a minimally invasive approach. Open exposure would also have required extensive dissection around the large, thrombosed sac. This sac lay directly adjacent to the tibial nerve and popliteal vein, and such dissection was expected to increase surgical morbidity. Contemporary guidelines and comparative studies support endovascular repair as a reasonable alternative in appropriately selected patients [4,9,10,11]. Panel (**C**) shows two overlapping self-expanding Viabahn covered stent grafts (white arrowheads) deployed after successful traversal of the occluded femoropopliteal segment. Together they exclude the aneurysm and restore arterial continuity. To support long-term vascular patency after Viabahn covered stent-graft placement, the patient received dual antiplatelet therapy comprising aspirin 100 mg/day and clopidogrel 75 mg/day for 6 months, followed by lifelong aspirin 100 mg/day monotherapy. This regimen was prescribed in accordance with current guideline recommendations for lower extremity peripheral artery disease [9]. Abbreviations: Dis., distal; Pro., proximal.

**Figure 5:**
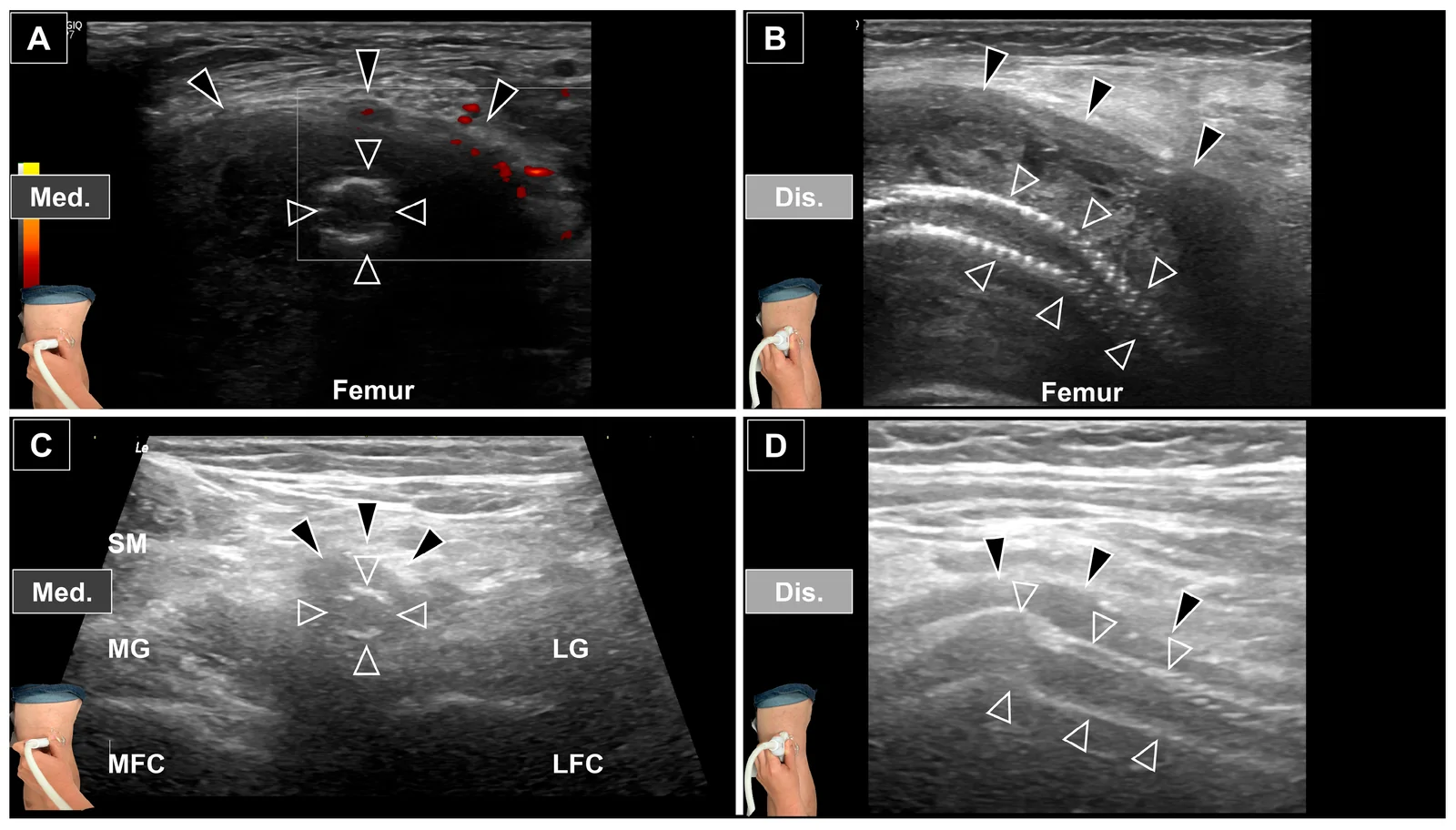
Serial ultrasonographic follow-up at three and 18 months following endovascular exclusion of a thrombosed popliteal artery aneurysm. Panels (**A**,**B**) show the short-axis and long-axis views at three months, whereas panels (**C**,**D**) show the corresponding views at 18 months. The white open arrowheads indicate the stent graft, which remains structurally stable and patent without sonographic evidence of re-occlusion [4,8]. The black arrowheads indicate the remaining aneurysmal sac with heterogeneous hypoechoic thrombotic content. Compared with the three-month examination, the 18-month surveillance shows progressive remodeling of the remaining aneurysm, with reduced thrombus burden and decreased mass effect on adjacent neurovascular and muscular structures [2,4]. Clinically, the preoperative right foot paresthesia improved progressively over the first 3 months after endovascular repair, in parallel with the sonographic findings. Secondary surgical decompression was not required. Decompression remains appropriate when neurological symptoms are attributable predominantly to persistent mass effect, as has been described for other peripheral arterial aneurysms [12,13]. In this patient, however, symptom relief followed both restoration of arterial inflow and progressive reduction in sac volume, and the relative contribution of chronic ischemia and local compression therefore cannot be separated. During long-term aspirin monotherapy, serial ultrasound at 18 months confirmed sustained stent-graft patency and progressive aneurysmal sac remodeling. Secondary interventions have been reported in approximately 33% to 63% of patients within two years of popliteal artery aneurysm repair [4], and long-term imaging surveillance is therefore warranted. Ultrasonography is well suited to this task, since it requires neither contrast nor ionizing radiation and can be repeated at the bedside to assess stent-graft patency, sac remodeling, and recurrent disease. By documenting both durable stent-graft stability and progressive aneurysm sac remodeling, this 18-month ultrasound follow-up complements the initial multimodal assessment and underscores the value of longitudinal imaging follow-up in this atypical presentation [5]. Abbreviations: Dis., distal; Med., medial.

## Data Availability

The original contributions presented in the study are included in the article; further inquiries can be directed to the corresponding author.

## Abbreviations

The following abbreviations are used in this manuscript:

ABI	Ankle-brachial index
Ant.	Anterior
BF	Biceps femoris
DSA	Digital subtraction angiography
DWI	Diffusion-weighted imaging
F	Femur
Lat.	Lateral
LG	Lateral gastrocnemius
Med.	Medial
MG	Medial gastrocnemius
MRI	Magnetic resonance imaging
PAA	Popliteal artery aneurysm
Post.	Posterior
SFA	Superficial femoral artery
SM	Semimembranosus
ST	Semitendinosus
STIR	Short tau inversion recovery
